# Distribution, trends, and antifungal susceptibility of *Candida* species causing candidemia in Japan, 2010–2019: A retrospective observational study based on national surveillance data

**DOI:** 10.1093/mmy/myac071

**Published:** 2022-09-12

**Authors:** Toshiki Kajihara, Koji Yahara, Minoru Nagi, Norikazu Kitamura, Aki Hirabayashi, Yumiko Hosaka, Masahiro Abe, Yoshitsugu Miyazaki, Motoyuki Sugai

**Affiliations:** Antimicrobial Resistance Research Center, National Institute of Infectious Diseases, 4-2-1 Aoba-cho Higashimurayama, Tokyo 189-0002, Japan; Antimicrobial Resistance Research Center, National Institute of Infectious Diseases, 4-2-1 Aoba-cho Higashimurayama, Tokyo 189-0002, Japan; Antimicrobial Resistance Research Center, National Institute of Infectious Diseases, 4-2-1 Aoba-cho Higashimurayama, Tokyo 189-0002, Japan; Department of Fungal Infection, National Institute of Infectious Diseases, 1-23-1 Toyama Shinjuku-ku, Tokyo 162-8640, Japan; Antimicrobial Resistance Research Center, National Institute of Infectious Diseases, 4-2-1 Aoba-cho Higashimurayama, Tokyo 189-0002, Japan; Antimicrobial Resistance Research Center, National Institute of Infectious Diseases, 4-2-1 Aoba-cho Higashimurayama, Tokyo 189-0002, Japan; Antimicrobial Resistance Research Center, National Institute of Infectious Diseases, 4-2-1 Aoba-cho Higashimurayama, Tokyo 189-0002, Japan; Department of Fungal Infection, National Institute of Infectious Diseases, 1-23-1 Toyama Shinjuku-ku, Tokyo 162-8640, Japan; Department of Fungal Infection, National Institute of Infectious Diseases, 1-23-1 Toyama Shinjuku-ku, Tokyo 162-8640, Japan; Antimicrobial Resistance Research Center, National Institute of Infectious Diseases, 4-2-1 Aoba-cho Higashimurayama, Tokyo 189-0002, Japan

**Keywords:** *Candida* species, candidemia, antifungal resistance, azole resistance, bloodstream infections

## Abstract

The increasing incidence of candidemia and the emergence of drug-resistant *Candida* species are major concerns worldwide. Therefore, long-term surveillance studies are required. Here, we provide one of the largest longitudinal overviews of the trends in the prevalence of *Candida* species using national data of 57 001 candidemia isolates obtained from > 2000 hospitals for the 2010–2019 period in the Japan Nosocomial Infections Surveillance database. The proportion of *Candida* species, except *Candida krusei* and *Candida guilliermondii*, was almost the same during the study period. The proportion of *C. guilliermondii* surpassed that of *C. krusei* in 2014. The incidence of candidemia due to *C. albicans* (*P *< 0.0001), *C. parapsilosis* (*P* = 0.0002), and *C. tropicalis* (*P *< 0.0001) have decreased significantly over this period. Azole susceptibility of *C*. *tropicalis* was low, with 17.8% of isolates resistant to fluconazole and 13.5% resistant to voriconazole. The micafungin susceptibility of *C. glabrata* was low, with 8.0% of isolates showing resistance. The resistance rate of *C. krusei* toward amphotericin B fluctuated considerably (between 3.2% and 35.7%) over this period. The incidence rate of candidemia caused by *C. parapsilosis* and *C. guilliermondii* in hospitals responsible for bone marrow transplantation was significantly higher than that in other hospitals. Overall, our study suggests that in Japan, the species distribution of *Candida* was almost the same in this period and similar to that reported in North America and Europe. A relatively high resistance to azoles and micafungin was observed in *C. glabrata*, *C. tropicalis*, and *C. krusei* isolates, which require continued surveillance.

## Introduction

Candidemia is one of the most common healthcare-associated bloodstream infections worldwide.^1^ It also remains a threat to susceptible patients and has a high crude 30-day mortality rate of approximately 30%.^2^^,^^3^ Previous studies have shown that patients with invasive fungal diseases incur additional costs, experience longer hospitalizations, and have higher mortality rates compared with those without such diseases, especially *Candida* infections which account for > 1.4 billion US dollars.^4^ The increasing population of *Candida* spp., along with decreasing azole susceptibility and emergence of echinocandin resistance are also major concerns.^5^

Previous studies from various countries have shown the epidemiology of candidemia. In the US, data of 1226 patients with candidemia from nine surveillance sites were collected in 2017, with an estimated incidence of 7 cases/100 000 persons. The most common *Candida* species was *C. albicans* (38%), followed by *C. glabrata* (30%), *C. parapsilosis* (14%), and *C. tropicalis* (7%).^6^ In Latin America, 672 candidemia cases that occurred in seven countries were collected from 20 surveillance sites during 2008–2010. The most common *Candida* species in this study were *C. albicans* (37.6%), *C. parapsilosis* (26.5%), and *C. tropicalis* (17.6%), whereas the distribution of *C. glabrata* was low.^7^ In China, 4010 *Candida* spp. were isolated from candidemia cases from 77 hospitals during 2015–2017. The most common *Candida* species among them was *C. albicans* (32.9%), followed by *C. parapsilosis* (27.1%), *C. tropicalis* (18.7%), and *C. glabrata* (12.0%).^8^ These previous studies indicate regional differences in the distribution of *Candida* spp. Meanwhile, the difference in antifungal susceptibility among *Candida* species was reviewed by Bassetti, et al.—the resistance rate of fluconazole (FLCZ) was found to be 0.6–1.7% in *C. albicans*, 8.0–16.2% in *C. glabrata*, 1.3–15.0% in *C. parapsilosis*, and 1.1–5.1% in *C. tropicalis* around the world.^9^

In Japan, a national surveillance, which spanned 105 participating hospitals, reported data for a single year, 2002.^10^ Another study reported chronological surveillance data from 10 participating hospitals between 2003 and 2014.^11^ However, this report did not take antifungal susceptibility into consideration. In this study, we utilized comprehensive surveillance data collected in a national antimicrobial resistance surveillance program—the Japan Nosocomial Infections Surveillance (JANIS)—in which all routine microbiological test results are being collected for all sample types from both symptomatic and asymptomatic patients from hundreds or thousands of participating hospitals since 2000.^12^ We evaluated the distribution trend, incidence rate, and antifungal susceptibility of *Candida* species using the data from JANIS for the 2010–2019 period.

## Materials and methods

### Data preparation and tabulation

All inpatient data fields from January 2010 to December 2019 were extracted from the JANIS database, which comprises results of all routine microbiological diagnostic tests (including both culture-positive and culture-negative results) and antimicrobial susceptibility testing results.^12^ In total, 2075 hospitals across Japan submitted their data to the JANIS database in 2019; these included 47 of 53 (88.7%) hospitals with over 900 beds, 291 of 355 (82.0%) hospitals with 500–899 beds, 1019 of 2174 (46.9%) hospitals with 200–499 beds and 718 of 5790 (12.4%) hospitals with < 200 beds. We used a Java toolkit to extract the aggregated data on the number of isolates of *C. albicans*, *C. glabrata*, *C. parapsilosis*, *C. tropicalis*, *C. krusei*, *C. guilliermondii*, *C. dublinensis*, and others that were isolated from blood samples and were subjected to antifungal susceptibility testing for FLCZ, voriconazole (VRCZ), and micafungin (MCFG) in accordance with the Clinical and Laboratory Standards Institute (CLSI) M60, 2017 criteria,^13^ along with antifungal testing for amphotericin B (AMPH) in accordance with the European Committee on Antimicrobial Susceptibility Testing (EUCAST) guidelines (Supplementary Table S1).^14^ The number of patients with candidemia was tabulated by focusing on hospitals responsible for bone marrow transplantation in 2019, using the JANIS database. The data were tabulated for each species using the deduplication algorithm used in the World Health Organization's Global Antimicrobial Surveillance System.^15^^,^^16^ To test whether hospitals responsible for bone marrow transplantation can be a high-risk group for candidemia, the data were also tabulated separately for such hospitals and others to compare the distribution and incidence of *Candida* species. The 186 hospitals were certified by Japanese Society for Transplantation and Cellular Therapy. We compared the data on hospitals responsible for bone marrow transplantation with data on other hospitals from 2019.

### Statistical analysis

The statistical significance of the differences in proportions was tested using Pearson's *χ*^2^ test or Fisher's exact test (when the minimum count in a contingency table was < 5). Cochran–Armitage trend test was used to test any trend in the incidence (i.e., number of candidemia cases divided by total number of patients who underwent blood culture testing) across years. The level of significance was set at *P *< 0.05. All statistical analyses were performed using the R software (version 4.0.5) and JMP Pro version 13 (SAS Institute, Cary, NC, USA).

### Ethical considerations

Patient identifiers were de-identified by each facility before submission to JANIS. Approval for the extraction and use of the data was granted by the Ministry of Health, Labor and Welfare of Japan (approval number: 0506–5).

## Results

### Distribution of *Candida* species isolated from blood samples from 2010–2019

The annual number of patients with candidemia and that of all patients from whom blood samples were collected from 2010–2019 are shown in Table 1. In this period, blood samples were collected from 6 821 580 patients, of which 55 580 were diagnosed with candidemia, and 57 001 *Candida* isolates were detected. The average age of the patients was 71.5 ± 17.0 years (Table 1).

**Table 1.** Distribution and trend of candidemia in Japan between 2010 and 2019

	Total	2010	2011	2012	2013	2014	2015	2016	2017	2018	2019
No. of participating hospitals		489	593	660	745	883	1435	1653	1795	1947	2075
No. of candidemia patients	55 580	2987	3558	4105	4482	4602	6544	7006	7161	7241	7894
No. of patients who underwent blood culture testing	6 821 580	307 341	385 031	436 886	490 472	555 128	824 998	900 862	933 528	980 392	1 006 942
Male sex	32 784	1811	2092	2429	2681	2701	3863	4105	4177	4257	4668
Female sex	20 921	1073	1324	1519	1591	1674	2475	2670	2769	2791	3035
Unknown sex	1875	103	142	157	210	227	206	231	215	193	191
Age (mean+-SD)	71.5 (±17.0)	69.6 (±17.9)	69.9 (±17.7)	70.1 (±17.9)	70.5 (±17.2)	70.6 (±17.2)	71.4 (±17.2)	71.8 (±17.0)	72.5 (±16.3)	72.3 (±16.7)	73.1 (±16.4)
Unknown age	2246	57	53	61	53	91	257	321	370	466	517
No. of isolates											
Total	57 001	3058	3632	4204	4596	4712	6730	7221	7341	7414	8093
*C. albicans*	24 864	1325	1665	1915	2009	2095	2955	3093	3069	3186	3552
*C. glabrata*	11 107	526	646	766	829	850	1275	1451	1579	1583	1602
*C. parapsilosis*	10 764	525	602	730	875	928	1252	1413	1426	1421	1592
*C. tropicalis*	3827	228	240	293	298	294	459	499	490	504	522
*C. krusei*	817	50	59	66	80	51	95	110	99	101	106
*C. guilliermondii*	949	51	51	55	61	85	127	123	119	122	155
*C. dublinensis*	82	0	3	3	4	2	7	9	20	14	20
Other	4591	353	366	376	440	407	560	523	539	483	544

**Figure 1.**
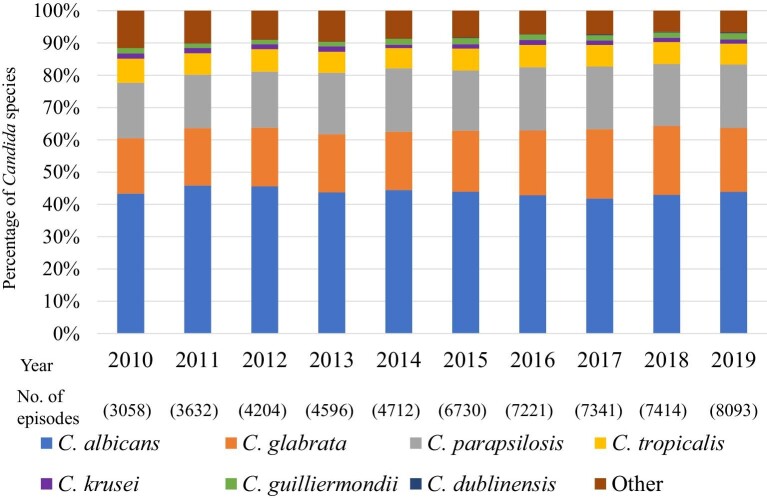
*Candida* species distribution during 2010–2019.

The distribution of *Candida* species isolated from the patients was as follows (Table 1): 43.6% (*n* = 24 864) *C. albicans*, 19.5% (*n* = 11 107) *C. glabrata*, 18.8% (*n* = 10 764) *C. parapsilosis*, 6.7% (*n* = 3827) *C. tropicalis*, 1.4% (*n* = 817) *C. krusei*, 1.7% (*n* = 949) *C. guilliermondii*, 0.15% (*n* = 82) *C. dublinensis*, and 8.1% (*n* = 4591) other. The distribution followed the similar trend from 2010–2019, except *C. krusei* and *C. guilliermondii* (Figure 1). The proportion of *C. guilliermondii* surpassed that of *C. krusei* in 2014. The proportion of *C. krusei* did not change significantly (*P* = 0.2103, from 1.65% in 2011–1.35% in 2019), whereas that of *C. guilliermondii* increased marginally significantly from 1.40% in 2011–1.92% in 2019 (*P* = 0.0613).

### Trends and incidence of candidemia

**Figure 2.**
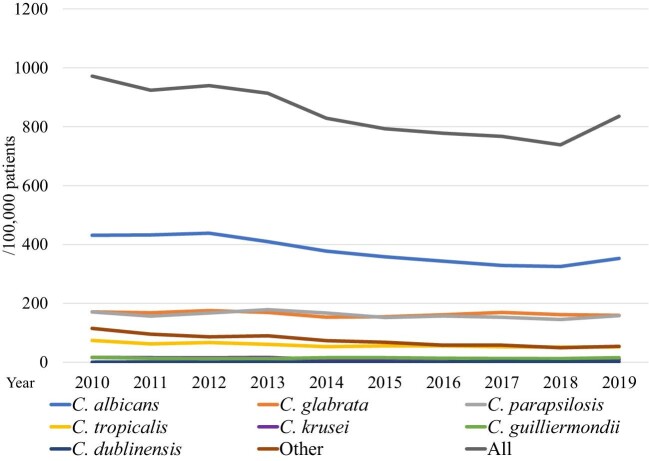
Annual incidence of nosocomial candidemia during 2010–2019.

The overall incidence of inpatient candidemia was 836/100 000 patients who underwent blood culture testing (Figure 2). The incidence of candidemia due to *C. albicans*, *C. parapsilosis*, and *C. tropicalis* has decreased from 2010–2019, with decreases of 18.2% (*P *< 0.0001), 7.45% (*P* = 0.0002), and 30.1% (*P *< 0.0001), respectively. On the other hand, that due to *C. dublinensis* has increased (*P *< 0.0001). The species-specific incidence was calculated for the entire study period, and blood cultures of 364/100 000 patients tested positive for *C. albicans*, 163/100 000 for *C. glabrata*, 158/100 000 for *C. parapsilosis*, 56/100 000 for *C. tropicalis*, 12/100 000 for *C. krusei*, 14/100 000 for *C. guilliermondii*, and 1.2/100 000 for *C. dublinensis*.

### Notable susceptibility patterns towards antifungal agents

**Figure 3.**
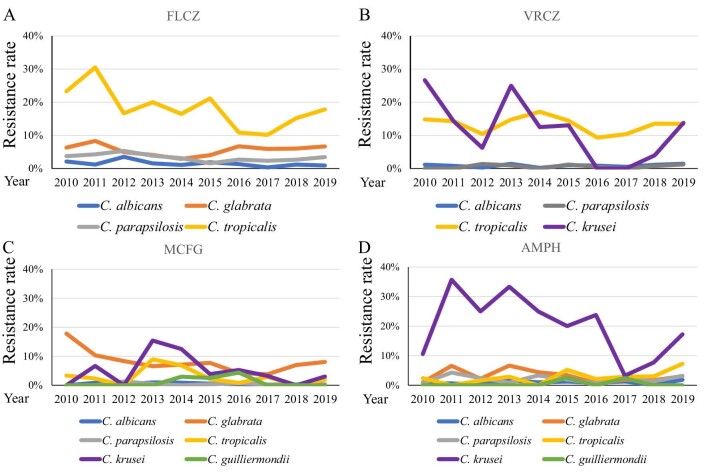
Antifungal resistance of each *Candida* spp. (A) Fluconazole (FLCZ), (B) voriconazole (VRCZ), (C) micafungin (MCFG), and (D) amphotericin B (AMPH). The following *Candida* spp. are shown**:***C. albicans* (blue line), *C. glabrata* (orange line), *C. parapsilosis* (gray line), *C. tropicalis* (yellow line), and *C. krusei* (purple line).

Only some of the isolates submitted to the JANIS database (29.2% [7247/24 854 isolates] of *C. albicans*, 28.2% [3137/11 107 isolates] of *C. glabrata*, 32.5% [3499/10 764 isolates] of *C. parapsilosis*, 28.9% [1107/3827 isolates] of *C. tropicalis*, 29.7% [243/817 isolates] of *C. krusei*, and 40.7% [386/949 isolates] of *C. guiliermondii*) were subjected to antifungal susceptibility testing. The trends of susceptibility of *Candida* spp. are summarized in Figure 3. *C. albicans* (blue line) and *C. parapsilosis* (gray line) isolates remained susceptible to all four antifungal agents (resistance < 6%) through the investigated period. The FLCZ resistance of *C. glabrata* (orange line) was < 9%. The MCFG resistance of *C. glabrata* was high, ranging from 8.0–17.8%. FLCZ and VRCZ were examined as representative antifungal agents of the azole class except that FLCZ was not used for *C. krusei* because of its intrinsic resistance. Azole susceptibility of *C. tropicalis* (yellow line) was low, with 17.8% of isolates resistant to FLCZ and 13.5% resistant to VRCZ in 2019. The resistance rate of *C. krusei* (purple line) to AMPH fluctuated considerably over the years, ranging between 3.2% and 35.7%. In 2019, 0.085% of *C. albicans*, 0.56% of *C. glabrata*, 0.17% of *C. parapsilosis*, and 1.12% of *C. tropicalis* isolates were cross-resistant to FLCZ and MCFG. Furthermore, 1.6% of the *C. krusei* isolates studied in 2019 were cross-resistant to VRCZ and MCFG.

### Comparison between hospitals responsible for bone marrow transplantation and other hospitals

**Figure 4.**
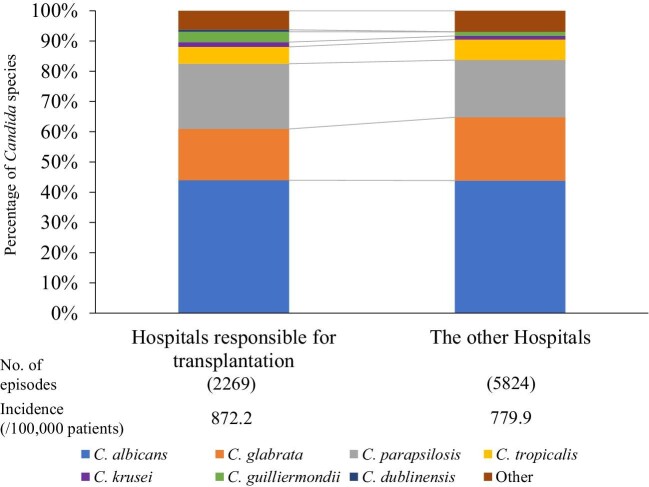
*Candida* species distribution stratified by hospitals responsible for bone marrow transplantation and other hospitals in 2019.

Candidemia often occurs in immunocompromised patients. We hypothesized that hospitals actively responsible for such patients and other hospitals could show differences in the distribution of *Candida* spp. The difference in the species distribution and incidence of *Candida* species between hospitals responsible for bone marrow transplantation and the other hospitals in 2019 is shown in Figure 4. The distribution of *Candida* species in hospitals responsible for bone marrow transplantation was as follows: 44.0% *C. albicans*, 16.9% *C. glabrata*, 21.6% *C. parapsilosis*, 5.6% *C. tropicalis*, 1.6% *C. krusei*, 3.4% *C. guilliermondii*, 0.7% *C. dublinensis*, and 6.3% other species. The distribution in other hospitals was 43.9%, 20.9%, 18.9%, 6.8%, 1.2%, 1.3%, 0.1%, and 6.9%, respectively. The proportion of *C. parapsilosis* and *C. guilliermondii* in hospitals responsible for bone marrow transplantation was significantly higher than that in other hospitals (*P *= 0.0072 and *P *< 0.001, respectively). Incidence of candidemia in hospitals responsible for bone marrow transplantation was higher than that in the other hospitals (872.2 vs. 779.9/100 000 tested patients who underwent blood culture testing). A comparison of the antifungal susceptibility of each *Candida* spp. In 2019 (Table 2) showed a significantly higher resistance rate for FLCZ in *C. parapsilosis* only in hospitals responsible for bone marrow transplantation than in the other hospitals (5.41% vs. 1.72%, *P *= 0.033). Because breakpoints for FLCZ were not defined in *C. guilliermondii*, the distribution of MIC values of FLCZ was compared between hospitals responsible for bone marrow transplantation and the other hospitals (Supplementary Figure S1); the percentage of strains with FLCZ MIC > 4 tended to be higher in the former (*P* = 0.056). Regarding MCFG, there were no significant differences in the resistance rate between hospitals responsible for bone marrow transplantation when compared to the other hospitals (Table 2).

**Table 2.** The resistance rate of each *Candida* spp. for antifungal agents in hospitals responsible for transplantation compared to other hospitals in 2019.

Species	Antifungal agent	Hospitals responsible for transplantation	Other hospitals	*P* value*
*C. albicans*	Fluconazole	1.04% (*n* = 477)	0.84% (*n* = 595)	0.974
	Voriconazole	0.99% (*n* = 406)	1.79% (*n* = 560)	0.450
	Micafungin	0.45% (*n* = 449)	0.17% (*n* = 599)	0.580
	Amphotericin B	1.46% (*n* = 410)	2.13% (*n* = 563)	0.601
*C. glabrata*	Fluconazole	9.09% (*n* = 176)	5.21% (*n* = 288)	0.152
	Micafungin	7.42% (*n* = 175)	8.39% (*n* = 310)	0.842
	Amphotericin B	2.38% (*n* = 168)	3.51% (*n* = 285)	0.697
*C. parapsilosis*	Fluconazole	5.41% (*n* = 259)	1.72% (*n* = 291)	0.033
	Voriconazole	1.74% (*n* = 230)	0.78% (*n* = 255)	0.430
	Micafungin	0.40% (*n* = 248)	0.36% (*n* = 279)	1.00
	Amphotericin B	3.11% (*n* = 225)	3.27% (*n* = 275)	1.00
*C. tropicalis*	Fluconazole	20.7% (*n* = 58)	16.2% (*n* = 99)	0.618
	Voriconazole	13.0% (*n* = 46)	13.7% (*n* = 95)	1.00
	Micafungin	0% (*n* = 54)	2.97% (*n* = 101)	0.552
	Amphotericin B	9.09% (*n* = 44)	6.45% (*n* = 93)	0.726
*C. krusei*	Voriconazole	12.5% (*n* = 16)	15.4% (*n* = 13)	1.00
	Micafungin	6.25% (*n* = 16)	0% (*n* = 17)	0.485
	Amphotericin B	20.0% (*n* = 15)	14.3% (*n* = 14)	1.00
*C. guilliermondii*	Micafungin	0% (*n* = 33)	0% (*n* = 25)	1.00
	Amphotericin B	0% (*n* = 28)	0% (*n* = 22)	1.00

*χ^2^ test or Fisher exact test

## Discussion

In this study, we evaluated the distribution, trend, incidence, and antifungal susceptibility of *Candida* species isolated from patients with candidemia in Japan, based on the national surveillance data from JANIS (2010–2019).

We show that there was almost no change in the distribution of *Candida* species isolated from the candidemia cases that occurred from 2010–2019 (Figure 1). In contrast, Lamoth, et al. reported that the distribution of *Candida* spp. has been changing; specifically, there has been a decrease in the proportion of *C. albicans* and an increase in that of *C. glabrata* and *C. parapsilosis*.^5^ In Japan, Kakeya, et al. analyzed 1921 *Candida* isolates from 10 hospitals between 2003 and 2014 and reported an increase in the proportion of non-*albicans* candidemia.^11^ In a related study, Pfellar, et al. reported that the proportion of *C. albicans* decreased to under 50% in 2009 in 39 countries, including Japan.^17^ Overall, these studies suggest that non-*albicans* candidemia in Japan has potentially increased between 2003 and 2009. The proportion of *C. guilliermondii* surpassed that of *C. krusei* in 2014. In many studies, the number of these *Candida* spp. reported was small. In a report from Switzerland^18^ and Denmark^19^ the distribution of *C. krusei* showed almost no change over 10 years, whereas there were no longitudinal data for *C. guilliermondii*. However, continued surveillance is required.

In a meta-analysis of various reports from European countries, Koehler, et al. reported that 40–50% of candidemia cases were caused by *C. albicans*, 10–30% by *C. glabrata*, and 2–15% by *C. parapsilosis*, which is similar to the results of the present study.^20^ Furthermore, similar results were reported in a study from Australia.^21^ However, contrary results were obtained in a national surveillance study in China that evaluated the proportion of each *Candida* species among 4010 candidemia isolates obtained from 77 hospitals from 2015–2017.^8^ According to this study, *C. albicans* accounted for 32.9%, *C. parapsilosis* for 27.1%, *C. glabrata* for 12.0%, and *C. tropicalis* for 18.7% of the isolates. These results were considerably different from our results as the proportion of isolated *C. albicans* was 10% lower, whereas that of isolated *C. parapsilosis* and *C. tropicalis* was 10% higher compared with that observed in our study. Another notable difference between previous studies in Asia and the present study was the proportion of *C. tropicalis*. Although it was 5.8% in the present study, Tan, et al. obtained *Candida* spp. From seven countries in the Asia-Pacific region and reported *C. tropicalis* to be the second most abundant isolate (30.7%).^22^ In India and Latin America, it has been reported that *C. tropicalis* is one of the most frequently encountered species.^7^^,^^23^^,^^24^

Overall, our study shows that the proportion of each *Candida* species isolated from candidemia cases in Japan is more similar to that in the US and Europe, than to the proportion in the Asia-Pacific region, which may be due to the differences in the climate. Japan lies between 20° and 46°N and is in line with Europe and North America. The proportion of *C. tropicalis* (yellow in supplementary Figure S2) in Okinawa (located in southern Japan) was significantly higher than that in Hokkaido (located in northern Japan; Pearson's *χ*^2^ test *P* ≤ 0.0001), suggesting a correlation between the climate (or climate-related factors) in southern regions and the increased proportion of *C. tropicalis*.

The annual incidence rate of candidemia caused by *C. albicans*, *C. parapsilosis*, and *C. tropicalis* showed a significantly decreasing trend (Figure 2). We could not show the incidence of candidemia in terms of population or the number of hospitalizations because the number of admissions is not a mandatory field in the voluntary-based JANIS database. Therefore, we used the number of patients who underwent blood culture tests to evaluate the incidence of candidemia. The overall incidence of candidemia over the 10 years was 0.83%, which was higher than that (0.58 ± 0.09%) reported by Kakeya, et al. The difference is possibly because this study used the number of patients who underwent blood culture tests, while Kakeya et al. used the number of blood cultures as the denominator to calculate the incidence rate.^11^ The number of blood cultures was greater than the number of patients who underwent blood culture tests. The incidence of candidemia caused by *C. dublinensis* significantly increased. The cause of this increase appears to be related to the development of species identification method, such as CHROMagar^TM^-Candida, API C oxanogram, the system for microorganism identification, and susceptibility testing (e.g., Walkaway^®^, BD phenix^®^) and/or matrix-assisted laser desorption/ionization time-of-flight mass spectrometry, which are generally used nowadays in hospital laboratories in Japan. However, we could not test it because information about how species identification is conducted at each hospital is not collected in JANIS.

We also revealed annual trends in antifungal resistance to FLCZ, VRCZ, MCFG, and AMPH from 2010–2019 (Figure 3). In Japan, MCFG is generally prescribed for candidiasis.^25^ MCFG was used from 2002 and caspofungin (CASPO) was used from 2012. In the JANIS database, the CASPO susceptibility data were only submitted for 111/8093 (1.37%) isolates in 2019. We, therefore, focused on MCFG susceptibility. In the present study, the resistance rate of *C. tropicalis* for azoles was the highest. Bassetti, et al. reported the resistance rate of *C. tropicalis* for FLCZ to be 1.1% and 5.1% in Latin America and North America, respectively.^9^ In the present study, the resistance rate of *C. tropicalis* for FLCZ (10.1–30.5%) was higher than that previously reported for other regions. The resistance rate of *C. tropicalis* for VRCZ was also high (9.3–17.1%). The resistance rate of *C. tropicalis* for VRCZ was reported to be 14%, 25.6%, and 16.7% in the Asia-Pacific region,^22^ China^26^ and Australia,^21^ respectively. In Europe, the resistance rate of *C. tropicalis* for VRCZ was 6.2%, 8.6%, and 9% in Norway,^27^ Denmark^19^ and Switzerland,^18^ respectively. These results suggest that this resistant strain might have spread in the Asia-Pacific region. As for echinocandins, *C. glabrata* showed approximately 8% resistance to MCFG, and the other *Candida* spp., excluding *C. krusei*, showed < 2% resistance (Figure 3C). Pfaller et al. reported that the resistance rate of *C. glabrata* for MCFG was 0.4%, 0.6%, 0%, and 2.8% in the Asia-Pacific region, Europe, Latin America, and North America between 2006 and 2016, respectively.^17^ The resistance of *C. glabrata* to MCFG might be higher in Japan than in other countries because MCFG, which was developed in Japan,^28^ is more frequently prescribed in Japan. We show that all *Candida* spp., except *C. krusei*, are susceptible to AMPH (Figure 3D). No resistant *C. krusei* strains have been reported in the Asia-Pacific,^8^^,^^21^ Switzerland,^18^ and Latin America;^7^ however, a Norwegian study reported 34.8% resistance of *C. krusei* to AMPH between 2004 and 2012.^27^ The number of *C. krusei* isolates was small. Further studies are required to study the factors and mechanisms underlying the resistance of *C. krusei* to antifungal agents.

We also show that the incidence of candidemia was higher in hospitals responsible for bone marrow transplantation compared with that in other hospitals. Hematologic malignancy is a risk factor for candidemia.^1^ In a prospective cohort multicenter study, Gamaletsou, et al. reported that *C. parapsilosis* accounted for 50% of hospitalized adult patients with hematologic malignancies.^29^ Patients subjected to bone marrow transplantation usually have a central venous catheter, and those subjected to oral and gastrointestinal mucosal disorders and neutropenia are treated using total parenteral nutrition, which is one of the risk factors for candidemia caused by *C. parapsilosis*.^30^ The resistance of *C. parapsilosis* to FLCZ in hospitals responsible for bone marrow transplantation was significantly higher than that in the other hospitals. The isolation rate of *C. guilliermondii* potentially associated with increased MIC values for FLCZ in *C. guilliermondii* (Supplemenatry Figure S1). Oral FLCZ is being administered to prevent candidiasis in patients with hematologic malignancy. Azole prophylaxis may be associated with the isolation rate of *C. parapsilosis* and *C. guillermondii*. MCFG breakthrough candidemia may be also associated with the isolation rate of *C. parapsilosis*: Kimura, et. al. reported that 14/30 cases of MCFG breakthrough candidemia in patients with hematological disorders were caused by *C. parapsilosis*.^31^

This study has several limitations. First, the JANIS is a voluntary surveillance. Therefore, the number of participating hospitals varies each year. Second, JANIS has not been collecting information about how species identification is conducted in each hospital. Third, JANIS has not been collecting strains, but has collected data on species, specimen, and antifungal susceptibility reported by the participating hospitals. Fourth, drug susceptibility tests are conducted only for approximately 30% of the strains isolated in the hospitals, and different methods of antifungal susceptibility testing are used by these hospitals.

## Conclusions

Despite the limitations of this study, it provides one of the largest longitudinal overviews using the national surveillance data of culturing and antifungal susceptibilities of 57 001 *Candida* isolates from over 2000 hospitals collected over 10 years. The proportion of each *Candida* species that we examined annually was almost the same from 2010–2019 and similar to that reported in North America and Europe. It might depend on geographic factors and the underlying disease groups and therefore, should be interpreted with caution. The resistance of *C. tropicalis*, *C. glabrata*, and *C. krusei* to azole and micafungin was high. Continuous nationwide surveillance for *Candida* spp., including antifungal susceptibility testing, needs to be conducted in the future.

## Supplementary Material

myac071_Supplemental_Files
