# Supplementary material for: Distribution, trends, and antifungal susceptibility of Candida species causing candidemia in Japan, 2010–2019: A retrospective observational study based on national surveillance data
Source: Med Mycol. 2022 Sep 12;60(9):myac071. doi: 10.1093/mmy/myac071 (PMC9521341; doi:10.1093/mmy/myac071)
Supplement: myac071_Supplemental_Files [file myac071_supplemental_files.zip › mm-2022-0053-File006.docx]

**Table S1. Minimum Inhibitory Concentration (MIC) breakpoints categories of antifungal agents for each *Candida* species**

| Antifungal agent | Species | MIC breakpoints categories μg/mL | Reference |
| --- | --- | --- | --- |
|  |  | R ≥ |  |
| Fluconazole | *C. albicans* | 8 | CLSI M60 |
|  | *C. glabrata* | 64 |  |
|  | *C. parapsilosis* | 8 |  |
|  | *C. tropicalis* | 8 |  |
| Voriconazole | *C. albicans* | 1 |  |
|  | *C. parapsilosis* | 1 |  |
|  | *C. tropicalis* | 1 |  |
|  | *C. krusei* | 2 |  |
| Micafungin | *C. albicans* | 1 |  |
|  | *C. glabrata* | 0.25 |  |
|  | *C. parapsilosis* | 8 |  |
|  | *C. tropicalis* | 1 |  |
|  | *C. krusei* | 1 |  |
|  | *C. guilliermondii* | 8 |  |
| Amphotericin B | *C. albicans* | 2 | EUCAST Version 10.0 |
|  | *C. glabrata* | 2 |  |
|  | *C. parapsilosis* | 2 |  |
|  | *C. tropicalis* | 2 |  |
|  | *C. krusei* | 2 |  |
|  | *C. guilliermondii* | 2 |  |

CLSI: Clinical and Laboratory Standards Institute. EUCAST: European Committee on Antimicrobial Susceptibility Testing
