# Supplementary figures and images for: Distribution, trends, and antifungal susceptibility of Candida species causing candidemia in Japan, 2010–2019: A retrospective observational study based on national surveillance data
Source: Med Mycol. 2022 Sep 12;60(9):myac071. doi: 10.1093/mmy/myac071 (PMC9521341; doi:10.1093/mmy/myac071)

Figure S2

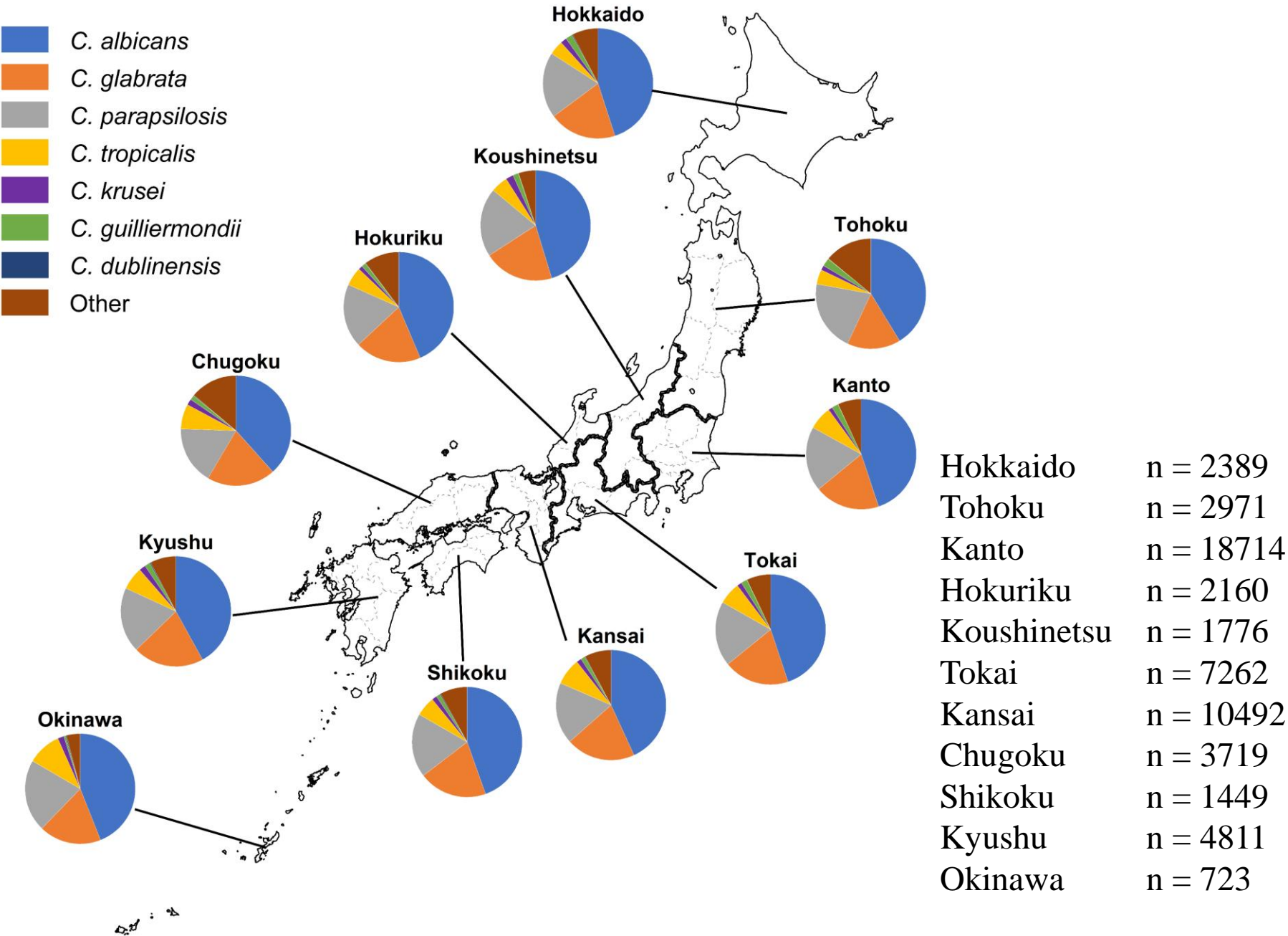

Supplement: myac071_Supplemental_Files [file myac071_supplemental_files.zip › mm-2022-0053-File008.pdf]

**Figure S1**

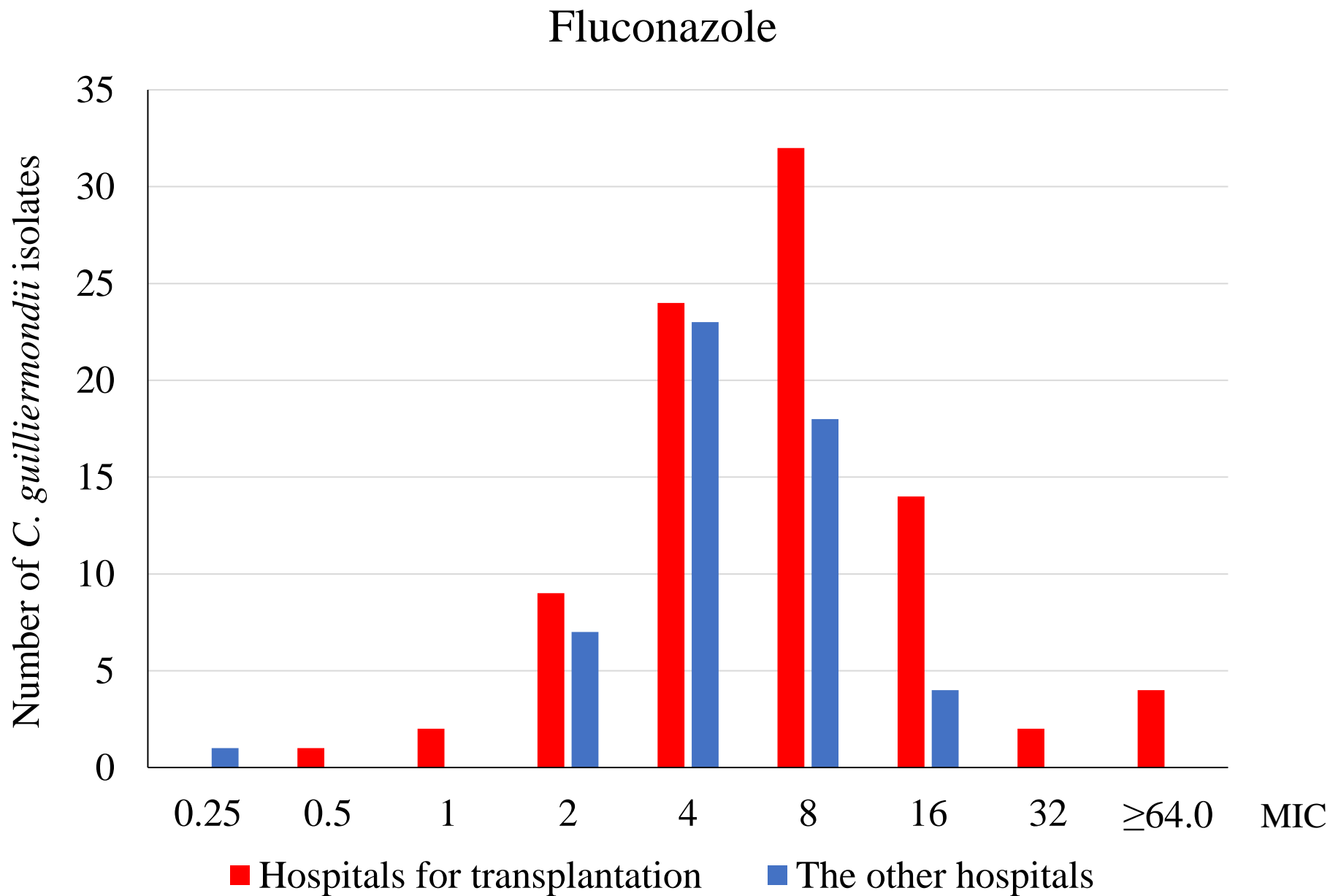

Supplement: myac071_Supplemental_Files [file myac071_supplemental_files.zip › mm-2022-0053-File007.pdf]
